# Supplementary material for: Are carbon emissions trading and green financial instruments synergistic? -Comprehensive quantitative research based on content analysis
Source: PLoS One. 2024 Mar 7;19(3):e0298601. doi: 10.1371/journal.pone.0298601 (PMC10919601; doi:10.1371/journal.pone.0298601)
Supplement: S1 Data — (ZIP) [file pone.0298601.s001.zip › Data/地级市Z_F工作报告.zip!/╜¡╬≈/╔╧╚─╩╨/└┤╫╘╣1⁄2╓┌║┼í╛┬φ┐╦╩2╛▌═°í┐_2015.docx]

各位代表：

现在，我代表市人民政府，向大会报告政府工作，请予审议，并请市政协委员和列席会议的同志提出意见。

一、2014年工作回顾

2014年是全面深化改革之年。在省委、省政府和市委的坚强领导下，全市上下紧紧围绕建设四省交界现代化区域中心城市和江西绿色崛起重要增长极的目标，认真落实“稳增长、促改革、调结构、惠民生”工作要求和“发展升级、小康提速、绿色崛起、实干兴赣”十六字方针，以全面深化改革为统揽，下大力气克服经济下行等不利因素影响，统筹做好各项工作，经济发展稳中有进，社会事业全面进步，较好地完成了市三届人大四次会议确定的目标任务。

一年来，主要做了以下工作：

（一）积极应对困难和挑战，经济保持较快发展

面对经济下行压力加大、一季度主要经济指标增幅下滑、财政收入出现负增长的严峻形势，坚持把稳增长放在首位，强化经济形势研判和经济运行的监测调度，主动跟进国家微刺激政策，用足用好省政府出台的“稳增长20条”措施，加强项目融资和对困难企业的帮扶，加大结构调整和招商引资力度，加快推进一批重大基础设施和保障性住房建设，以“组合拳”的方式实施精准发力，迅速扭转了经济下滑被动局面，主要经济指标实现了逐季攀升。全年完成财政总收入262.6亿元，增长13.2%，其中公共财政预算收入194.2亿元，增长18.1%，提前一年完成了“十二五”财政增收目标。预计全年生产总值1550亿元，增长10.5%；规模以上工业增加值653亿元，增长12.1%；500万元以上项目固定资产投资1374.3亿元，增长18%；社会消费品零售总额550亿元，增长13.5%；外贸出口总额36亿美元，增长18.3%。

（二）深入推进重点领域改革开放，发展动力和活力进一步增强

制定出台了《关于贯彻落实中央和省委全会精神全面深化改革的实施意见》及27个配套改革文件，在八个领域37个方面推出了217项改革。大力推进行政审批制度改革，市本级行政审批项目由原来的328项减到98项，精简率达70.1%，办理时限平均缩短60.7%。全面实行注册资本认缴登记制，新登记注册市场主体3.86万户，增长19.2%。稳步推进财税金融体制改革，启动了综合治税信息资源共享平台建设，完成了上饶农村商业银行的改革，德兴市成立了全省第1家民间借贷规范管理服务中心。完成了市公交公司国有国营体制改革，组建了江西现代路桥集团。全面完成了29个国有林场改革。扎实推进了农村土地承包经营权、农村集体土地所有权、宅基地和集体建设用地使用权确权登记发证工作。全面启动了市县政府职能转变和机构改革。 全面落实省政府《关于支持赣东北扩大开放合作加快发展的若干意见》。与南昌市就鄱余万三县融入南昌核心增长极战略进行了对接，与泰国暖武里省、芬兰福尔萨市签署了友好城市意向书，与贵州省遵义市缔结为友好城市。南昌海关驻上饶办事处升格为上饶海关。上饶—宁波“五定班列”升级为“海铁联运天天班”。

举办了浙江招商引资活动周、海内外侨商上饶行等19场重大招商推介活动，市、县两级主要领导共外出招商320批次。大力实施“饶商回归”工程，全市“饶商回归”投资项目超过150个，投资总额达356.6亿元。全市实际利用外资8.4亿美元，增长10.8%，其中现汇进资2.2亿美元，增长175.5%；利用省外5000万元以上项目实际进资438.3亿元，增长18.4%。 （三）围绕做强支柱产业调结构，加快了三次产业升级步伐

全市开工、在建投资亿元以上工业项目167个，列入全省重点调度项目6个，工业投资占全部固投的比重达54.8%。扶持了晶科能源、光电高科、星汉光学、凤凰光学战略重组等项目，强化了我市国家级光伏、光学产业基地的地位。引进了蓝途汽车、上菱电梯、逸源机电等项目，提升了我市先进装备制造业水平。推进了岩瑞铜业、远翔实业等一批新型环保节能回收一体化铜加工项目和中材机械、耐普矿机、艾芬达卫浴等一批老企业技改扩能项目，促进了有色金属产业和传统产业的技术升级。完成了广丰卷烟厂技改搬迁项目选址。4个工业产业集群列入全省重点发展产业集群。启动了经开区蓝途汽车产业园、饶商回归产业园、总部经济产业园、再生干细胞产业园“新四园”建设。完成了万年县、铅山县、德兴市工业园调园扩区，新增园区面积18平方公里，工业园区实际开发面积达90.2平方公里，列全省第二。预计全市工业园区主营业务收入2450亿元，增长8%。工业四大主导产业完成增加值458亿元，占全市规模以上工业的比重超过70%。 粮食总产突破70亿斤，实现“十一连增”。新建全国绿色食品原料标准化生产基地9个。万年县入选国家级现代农业示范区，并荣获“全国农业产业化示范基地”称号，德兴市红花茶油示范基地填补我省“全国有机农业示范基地”空白。10个农业产业集群列入全省重点发展产业集群。创建省级现代农业示范区6个，新增农业类中国驰名商标2个、“三品一标”农产品35个，新增省级农业龙头企业10家、农民专业合作社1361家。全市家庭农场达961家，农家乐超过2.3万家。

新创建国家4A级景区5个、国家级水利风景区5个，我市成为全省世界遗产、5A级景区、4A级景区数量最多的设区市。完成了灵山、大茅山、神农源等国家级新景区一期开发并正式对外开放。推进了龟峰景区扩建提升、婺源“梦里老家”山水实景演出、铅山河口古镇保护性开发、鄱阳世华城旅游商业区等一批重点旅游项目建设。我市被确定为第三批国家旅游标准化试点城市，三清山被评为第二批国家旅游标准化示范景区，婺源县入选国家生态旅游示范区，鄱阳湖国家湿地公园被全球自然基金会授予“世界生命湖泊最佳保护实践奖”，铅山县河口镇被国家认定为“万里茶道第一镇”。成立了市旅游发展委员会，牵头成立了赣东北旅游合作联盟，建立了中国旅游研究院全国首家乡村旅游观察点。举办了“金驹奖”2014世界大学生摄影展，为沪昆、合福高铁动车冠名了“上饶号”。加快了智慧旅游平台建设。全年接待游客7018.9万人次，旅游综合收入572.7亿元，两项指标继续保持全省领先。

建立省级电子商务示范基地3个，引进电商企业超过1000家，预计全年电子商务交易额达160亿元。信州区打造了上饶慧谷、上饶电商谷、淘宝园，玉山县、广丰县被列入“全国电子商务进农村综合示范试点县”，玉山县被评为“中国电子商务创新示范城市”。加快推进了全国中小商贸流通企业服务体系建设试点城市项目，中国南方棉布批发市场被评为首批“江西省重点商品交易市场”，鄱阳湖商贸批发中心被评为省级现代服务业集聚区，婺源县朱子步行街成为全省第一条“中国特色商业街”。开工建设了万达广场、万力时代、星河国际、中商广场、五洲国际商业城等一批大型城市商业综合体和万年国际珍珠城、鄱阳湖生态水产品交易中心、红都国际商贸中心、蓝丰建材商贸城等一批专业市场。

新增证券营业部4家，总数达8家；开业、筹建村镇银行9家，全市银行业金融机构达20家，列全省第二。德兴市城投公司获国家发改委核准发行8亿元企业债券；晶科能源增发了3亿美元股票和债券，并获2.25亿美元私募债投资；江西远泉股份有限公司在新三板挂牌交易。大力推广了“财园信贷通”、“财政惠农信贷通”、“助保贷”、“旅游贷”等新的融资方式，为实体经济发展注入了活力。全市银行业金融机构人民币存款余额1906.4亿元，增长14.5%，增幅列全省第一；贷款余额1234.1亿元、列全省第三，增长22.8%、列全省第二。

（四）加快重大基础设施建设，统筹推进城乡发展

完成了三清山机场航站楼主体土建及场道地基工程，建成了全国首个两条高铁骑跨式枢纽站。杭南长高铁正式运行，合福高铁铺轨贯通，九景衢铁路上饶段全线施工。万年港综合码头建成运营。德上高速三清山连接线、沪昆高速经开区互通及连接线、上武高速铅山连接线建成通车。德上高速怀玉山连接线和大茅山连接线加快建设，上万高速完成招标工作。硬化农村公路1784.6公里，新建、改造桥梁27座。大力推进路网规划调整，新增国道4条、省道17条。国电黄金埠电厂二期核准前期工作收尾，上饶火电厂完成初可研。横峰县100MW地面光伏电站项目一期完工，万年县建成了全国最大的综合利用沼气发电工程。改造中低产田15.6万亩，建设高标准农田7.9万亩，完成水利投资25.2亿元。	编制完成了上饶空港新区、城市景观风貌特色、消防等规划，启动了上饶市城市总体规划修编及高铁新区、西货站物流园区、综合交通、地下空间利用等规划编制工作。加快推进了中心城区老火车站城市综合体、新火车站综合交通枢纽、凤凰大道东延伸段、信江南岸河堤改造、上饶师范学校迁建等重点项目。推进了320国道上饶城区段改建、上广和上铅快速通道建设，初步形成了信江河谷城镇群“半小时经济圈”。中心城区拆除违法建筑10万平方米、拆除户外广告7000余块，有效净化了城市空间。投入建设资金6.5亿元建设了50个镇村联动点，新增镇村联动集镇面积30.6平方公里，17个镇被列入全国重点镇；全面完成了1425个新村点建设任务。	深入推进鄱阳湖生态经济区建设，深入推进原中央苏区振兴发展。通过积极争取，全境纳入长江中游城市群一体化发展规划，并成功争取到“宽带中国”示范城市和信息惠民国家试点城市。4G和政务网络覆盖乡镇。广丰县成功获批“中国建筑之乡”。德兴市、上饶县财政收入分别突破30亿元、20亿元大关。	（五）切实保障改善民生，社会事业全面发展，生态保护力度加大，社会和谐稳定

社会保险覆盖面不断扩大，城乡低保、企业退休人员基本养老金等保障水平稳步提高。新增城镇就业8.9万人，新增转移农村劳动力23.4万人，我市被农业部选定为全省唯一的新型职业农民培育整体推进试点市。在全省首创并全面启动城镇基本医疗保险大病关爱行动。全面推行高龄补贴制度，为70周岁以上老年人办理了免费乘车意外伤害保险。中心城区城西公交综合服务中心建成投入使用，新增新能源公交车90台。落实贫困村村庄整治项目722个，投入扶贫资金1.29亿元；建设移民集中安置点26个，搬迁移民1144户5204人。解决了39.6万农村人口饮水安全问题。开工建设各类保障性住房5.1万套，建成2.7万套。继2009年之后，我市再次荣获“中国幸福城市”称号。预计全市城镇居民人均可支配收入增长10%，农村居民人均可支配收入增长11.5%。

全市投入城镇生活污水处理整改资金达2.3亿元，年处理12万吨的上饶市污水处理厂西迁改造项目建成并投入使用。四项主要污染物年度减排目标和104个减排项目顺利完成。查处环境违法企业214家，其中取缔关停97家。再生资源循环经济产业园“一市场四中心”主体功能项目开工建设，已建和在建再生资源回收网点286个。我市被确定为全国首批四个负氧离子监测点之一。完成造林面积31.2万亩。中心城区成功创建省级森林城市，并启动了国家森林城市创建工作。广丰县铜钹山自然保护区成功晋级国家自然保护区，婺源入选第二批全国生态文明示范工程试点县，德兴、广丰获“国家珍贵树种培育示范县市”称号。

新建干细胞、鑫星农牧2个院士工作站。新增国家高新技术企业9家，总数达38家。组建了江西晶科光伏科技协同创新有限公司，实现了我市省级协同创新体零的突破。完成了1035所农村义务教育学校达标建设。德兴市、婺源县义务教育均衡发展工作通过国家和省督导评估，横峰县、弋阳县通过省政府教育督导评估。上饶师范学院成立了江西省塑料制备成型重点实验室，江西医专通过省高职高专人才培养工作水平评估，婺源茶校完成新校区建设并与江西农大开展合作办学。原创廉政诗画剧《为了可爱的中国》、原创赣剧《那杆秤》以及我市参与摄制的30集电视连续剧《油菜花香》、电影《桃子的爱情》获得好评。婺源县被评为“全国文化先进县”。婺源县、广丰县被评为“中国民间文化艺术之乡”。铅山县河口镇、石塘镇被评为中国历史文化名镇。万年稻作习俗、婺源绿茶制作技艺被列入第四批国家级非物质文化遗产项目名录。《上饶地区志（1991-2000）》付印出版。启动实施了广播电视直播卫星“户户通”工程，提前一年实现县县都有数字影院。举办了中华龙舟大赛、国际冬泳邀请赛、全国半程马拉松邀请赛等赛事活动。加快城乡医疗卫生服务体系建设，在3个县开展了县级公立医院改革试点，在全省率先启动新农合大病保险工作并实行了市级统筹。通过了省级卫生城市复检。落实了单独两孩政策，全市人口出生率、出生人口性别比分别下降1.76个千分点和1.6个基点。进一步加强了国防动员和民兵预备役工作。

加强重点行业和领域安全生产专项整治，全面推进防震减灾工作，安全生产形势平稳。创新社会管理，建立健全了信访事项第三方评议和调解机制，我市代表江西在全国信访督查工作会上作了典型发言。全面推进了基层应急管理规范化建设工作。加强食品药品安全监管，努力保障人民群众饮食用药安全。扎实推进“天网”、“地网”等治安防控体系建设，8类暴力犯罪发案率降幅达34.8%，降幅全省第一。

民族宗教、涉台事务、人民防空、气象水文、检验检疫、妇女儿童、新闻出版、社科研究、群众性精神文明创建、无线电管理、红十字、档案、保密、质监、石油、烟草、盐业、慈善、统计等各项事业取得新成绩。

（六）深入改进作风，政府自身建设得到加强

深入开展党的群众路线教育实践活动，有效治理了政府系统形式主义、官僚主义、享乐主义和奢靡之风。严格执行中央“八项规定”和国务院“约法三章”，三公经费下降24%。集中开展了“十大整治”活动。强化了审计监督和行政监察，严肃查处了一批损害群众利益的突出问题，严肃查办了一批违纪违法腐败案件。

严格按照法定权限和程序行使权力、履行职责，自觉接受市人大及其常委会的法律监督、工作监督和市政协的民主监督，主动向人大常委会报告工作，认真接受人大代表询问和政协委员意见建议，按期办复人大代表建议77件、政协委员提案198件，办结率100%，满意率98.9%。推进政府信息公开，主动公开信息28.6万条。全面推行政府法律顾问制度。荣获“全国‘六五’普法中期先进城市”称号。	各位代表，过去一年，我们克服种种困难和挑战，顶住经济下行压力，成绩来之不易！这是认真贯彻党中央、国务院一系列重大方针政策的结果，是省委、省政府和市委正确领导的结果，是全市干部群众团结奋进、共同努力的结果。在此，我代表市人民政府，向全市广大工人、农民、知识分子、干部和历任老领导、老同志，向各民主党派、工商联、无党派和社会各界人士，向驻饶人民解放军、武警官兵和公安民警，向中央和省驻饶单位及所有关心、支持上饶发展的同志们、朋友们，向在我市创业的投资者、建设者，致以崇高的敬意和衷心的感谢！	回顾过去一年工作，我们清醒地看到，经济社会发展中还存在不少问题。经济下行压力加大，企业融资更加困难；产业层次不高，创新能力不强；工业品出厂价格持续下降，要素成本不断上升；民生刚性支出需求增大，财政收支矛盾十分突出；区域竞争激烈，保位、进位压力越来越大；部分政府工作人员的思想仍然不够解放，责任意识、担当精神、工作作风与实干兴赣的要求不相适应。对于这些问题，我们一定高度重视，并采取有效措施加以解决。	二、2015年工作安排

2015年是全面深化改革的关键之年，是全面推进依法治国的开局之年，是全面完成“十二五”规划的收官之年，也是上饶全面进入高铁时代的第一年。今年全市经济社会发展的总体要求是：全面贯彻党的十八大、十八届三中、四中全会和中央经济工作会议精神，以邓小平理论、“三个代表”重要思想、科学发展观为指导，深入学习贯彻习近平总书记系列重要讲话精神，深入落实省委“发展升级、小康提速、绿色崛起、实干兴赣”十六字方针，紧紧围绕市委三届六次全会提出的发展目标和战略部署，咬定与全国全省同步全面建成小康社会的战略目标，全面深化改革，全面推进法治上饶建设，适应新常态，抢抓新机遇，展现新作为，争当生态文明标兵、绿色崛起先锋，推动全市经济社会持续平稳健康发展，努力完成“十二五”规划目标任务，奋力迈出建设四省交界现代化区域中心城市和江西绿色崛起重要增长极的新步伐。	今年全市经济社会发展主要预期目标是：生产总值增长9%左右，财政总收入增长12%以上，公共财政预算收入增长13%以上，规模以上工业增加值增长11%以上，500万元以上项目固定资产投资增长16.5%，社会消费品零售总额增长12.5%，外贸出口总额增长7%左右，实际利用外资增长10%，城镇居民人均可支配收入增长10%左右，农村居民人均可支配收入增长11%以上，人口自然增长率控制在8‰以内，居民消费价格总水平涨幅控制在3%左右，城镇登记失业率控制在4.5%以内，节能减排完成省政府下达的计划任务。	需要说明的是，今年全市经济发展主要预期指标，是按不低于全省计划增长水平来确定的。在实际工作中，我们将按更高的标准和要求来推进各项工作，力求发展更快一些。	做好今年经济社会发展工作，从总体上要把握好以下几点：一是把握“发展升级、小康提速、绿色崛起”的总体要求，始终保持较高的增长速度，不断提高经济发展的质量和效益，打造江西绿色崛起重要增长极。二是把握全面深化改革和扩大对外开放合作的内在规律，积极参与长三角、海西经济区等沿海发达地区分工合作，争当全省改革开放“先行者”。三是把握我省建设生态文明先行示范区上升为国家战略这一重大机遇，不断加强生态建设、巩固生态优势，打造“美丽上饶”。四是把握发展成果惠及全体人民的根本目标，加强社会保障，改善民生事业，建设更加平安、和谐、富裕的“幸福上饶”。五是把握法治政府建设的深刻内涵，依法全面履行政府职能，着力优化经济发展环境，全面激发社会创造活力。六是把握风险发生发展趋势，强化风险防控，建立风险预警和化解机制，防止经济惯性下滑，坚决守住风险底线。	重点做好八个方面的工作：

（一）以加快政府职能转变为核心，强力推进重点领域改革，努力在打造全省体制机制创新高地上取得新突破

加快行政管理体制改革。加快转变政府职能，全面完成市、县政府机构改革。在承接好国家和省下放审批事项的同时，再取消和下放一批市级审批事项。全面清理非行政许可审批，大幅度减少前置审批。深入推进“三单一网”改革，完成市县权力清单、责任清单、负面清单公布工作，加快建设全市网上审批系统，实现所有事项全流程、并联式上网公开审批。加快事业单位分类改革。深化行政执法体制改革，开展综合执法试点，大幅减少市县两级政府执法队伍种类，切实解决好多头执法、多层执法、随意执法等突出问题。加快行政机关及参公事业单位公务用车制度改革。

深化财税金融体制和市场化改革。改革完善预算管理制度，建立统一规范的部门预算体系，清理规范财税优惠政策，将政府债务分门别类纳入预算管理。将“营改增”范围扩大到建筑业、房地产业、金融业和生活服务业等领域，全面完成“营改增”任务。进一步扩大政府购买公共服务试点范围。加快农村商业银行的组建步伐，实现村镇银行县域全覆盖。深化政府融资平台改革，降低资产负债率，增强融资能力，提高投资效益，防范债务风险。进一步放宽市场准入，大幅度减少限制类条目。深化工商登记制度改革，减少工商登记前置行政许可事项。深化内贸流通体制改革，建设法治化营商环境。在公共服务领域再推出一批项目向社会资本开放。推进社会信用体系建设，在全市各工业园区推开小微企业信用体系建设。

深化国资国企改革。有序推进经营性国有资产集中统一监管。大力发展混合所有制经济，推动国有企业加快建立和完善现代企业制度，提高国有企业经营管理水平。完善国有资本经营预算制度，加强国有资本收益和使用管理。

推进城乡协调发展体制改革。推进户籍制度改革，加快制定与城镇化发展相适应的社保、教育、医疗、保障性住房等配套政策。稳步推进征地制度、农村宅基地管理制度和集体经营性建设用地入市改革工作。抓好农村土地承包经营权确权登记颁证工作。推进土地流转规范化服务试点，探索建立农村土地流转新机制。继续深化林权改革，积极推进林地流转。

（二）以推动高铁经济试验区上升为省级重大发展战略为目标，加速融入东南沿海，努力在打造全省对外开放合作高地上取得新突破

努力构建对外开放合作新格局。迎接高铁时代，建立“上饶高铁经济试验区”，并积极推动“试验区”上升为省级重大战略。充分发挥上饶“无水港”作用，利用宁波港、宁德港两个出海口，对接“一带一路”重大战略和福建自贸区建设，积极参与海上丝绸之路和海西经济区分工合作。加强与长三角经济区的对接合作，争取我市成为全省第一个长三角城市经济协调会议成员城市。加强与长江中游城市群规划对接，争取将我市 “1+5”信江河谷城镇群建设列入专项规划。

强力抓好招商引资。认真落实重大项目招商引资三年攻坚计划，瞄准国际知名企业、国内龙头企业，引进一批投资规模大、带动力强的产业项目。充分发挥商会平台作用，继续大力实施“饶商回归”工程。深入推进“央企入饶”。建立市县联动、部门合作的开放招商机制，提升招商工作效果。狠抓重大招商活动签约项目跟踪督导落实，确保招商取得实效。坚持本地民营企业和外来招商企业同等待遇，坚持“非禁即入”，着力营造公平公正的市场环境。全年力争新引进省外5000万元以上项目资金增长14%。

积极做好外经外贸工作。实施“走出去”战略，扩大对外承包工程，推动优势企业在国外投资办厂。加强外贸出口基地和出口品牌建设。

（三）以经开区和工业园区为主战场，坚定不移主攻工业，努力在打造全国重要的光伏、光学和先进装备制造业生产基地上取得新突破

继续做大园区平台。积极推进调园扩区，大力提升工业园区的承载能力。加快创建生态园区、智能园区，鼓励发展“飞地园区”。加快蓝途汽车、饶商回归、总部经济、再生干细胞“新四园”建设，推动上饶经开区尽快做大做强。大力提高工业园区的土地利用率和产出率。力争新增2个主营业务收入过200亿元的工业园区。

做大做强支柱产业。坚持把光伏产业作为上饶工业的“首位”产业来抓，支持晶科能源实施“三年倍增”计划，加快光伏新能源应用步伐，建设国家光伏新能源示范城市。坚持把光学产业做大做强，支持凤凰光学、星汉光学等光学企业的发展，积极打造“中国光学城”。支持蓝途汽车发展，重振上饶汽车产业。支持干细胞生命科学新兴产业发展，打造中国干细胞生命科学研发应用基地。支持新材料产业、生物医药产业加快发展。

改造提升传统优势产业。坚持有色金属主导产业地位不动摇，加快铜冶炼加工企业升级改造。加快德兴、铅山化工产业和弋阳、玉山、万年建材产业升级改造。加快信州苎麻纺织产业、广丰红木产业、德兴黄金产业、鄱阳硬质合金工具产业、余干水钻产业、横峰葛产业等传统优势产业集群发展。

强化企业在技术创新中的主体地位。实施创新驱动核心战略，发挥政府科技投入的引导作用，以企业主导实施产业科技项目，重点帮扶晶科能源申报省级知识产权运用示范企业，力争全年新增高新技术企业10家，申报国家中小企业技术创新基金项目20个，新增省级工程技术研究中心3家，培育创建创新团队5个。加强科技人才引进和培养，充分调动科技人员的积极性和创造性。

（四）以现代农业示范园区建设为重点，加快转变农业发展方式，努力在打造面向东南沿海的优质农产品种养加工基地上取得新突破

加快推进农业现代化。加强耕地保护和设施农用地管理，全面开展永久基本农田划定工作。加大高标准农田建设力度，按照“稳面积、攻单产、调结构、提品质”的要求，提升粮食综合生产能力。按照“有规划、有标准、有主体、有产业、有品牌、有机构”的要求，每个县创建1个以上农业示范园区。继续抓好“财政惠农信贷通”扶持政策落实，多渠道促进农民增收。深入推进以“生态鄱阳湖、绿色农产品”为主题的品牌发展战略，扶持一批优势企业创评国家级农业龙头企业。围绕建设全省现代农业强市的目标，加快农业产业化“1211”工程建设步伐，力争新增农民专业合作社300家、家庭农场200家、规模以上农业龙头企业20家，市级以上龙头企业销售收入突破300亿元。

加快建设美丽乡村。坚持全域规划、镇村联动、集中整治，高标准抓好1400个左右新农村点建设。严格镇村建设管理，下大力气规范农民建房。加大历史文化名镇名村和传统村落、传统民居保护力度。建立健全长效机制，巩固和扩大农村环境卫生集中整治成果。

（五）以旅游业为龙头，全面推动现代服务业发展，努力在打造全国重要旅游目的地上取得新突破

把旅游业打造成绿色崛起的先锋产业。实施旅游产业差异化发展战略，加快推进婺源、三清山、龟峰、铅山、鄱阳5个全省重点旅游产业集群发展和三清山—港首—婺源国际旅游度假区建设。继续支持三清山创建全省旅游示范景区、婺源创建全国旅游第一强县、鄱阳湖国家湿地公园创建国家生态旅游示范区和中国湖泊旅游休闲度假目的地。推进龟峰、篁岭创建国家5A级景区，灵山、葛仙山、铜钹山、云碧峰创建国家4A级景区。加快市旅游集散中心等中心城区景区、景点建设，做旺中心城区旅游。坚持高起点、高标准，支持各地、各景区新建、续建一批重大旅游项目，打造特色旅游增长点。推进国家旅游标准化试点城市和国家智慧旅游试点城市建设。加强旅游宣传和促销工作，成立高铁旅游联盟。办好全省旅游产业发展大会、第四届“万里茶道”与城市发展（铅山）中蒙俄市长峰会。办好婺源国际乡村旅游文化节、三清山国际旅游文化节、“金驹奖”国际摄影大赛等活动。力争全年接待游客8500万人次，旅游综合收入达到750亿元。

推动新兴服务业发展升级。出台《上饶市加快发展服务业三年行动计划（2015-2017年）》，加快建设现代服务业集聚区。推动电子商务加快发展。积极争创国家电子商务示范城市。全面实施“电子商务进农村”工程，培育20个左右的“电子商务村”。加快建设中国邮政（上饶）电商示范园，推进农村“E邮工程”。加快城乡商贸市场建设。继续抓好万达广场等一批大型城市综合体和大型专业市场建设。创新实施“万村千乡市场工程”，争创3个以上“江西省商贸强镇”。推动现代物流业加快发展。加快建设上饶（海西）物流城、中兵工物流金融等一批重点示范物流园区和物流港，鼓励制造业企业与第三方物流深度合作、联动发展。积极推进云计算、大数据、物联网等新兴信息服务业态。培育发展健康养生、文化创意、月光经济等产业。

推动金融保险业加快发展。加大金融机构引进力度，争取更多的商业银行来饶设立分支机构。进一步拓宽融资渠道，扩大融资规模，鼓励金融机构加大对实体经济特别是中小微企业的扶持。加大直接融资力度，积极推动鑫星农牧、耐普矿机等企业到主板上市，推动优质企业到全国中小企业股份转让系统挂牌融资。大力发展互联网金融。加大预防和打击非法集资活动的力度，推广设立民间借贷登记服务中心。发展现代保险业，加快推进保险资金直投工作。力争全年新增银行贷款235亿元，新增存款285亿元。

（六）以重大基础设施建设为支撑，做大做强中心城市，努力在打造四省交界现代化区域中心城市上取得新突破

加快推进重大基础设施建设。力争三清山机场主体工程10月份竣工、合福高铁6月底前正式运行。大力推进九景衢铁路、上饶西货站、上饶高铁存车场建设。加快宁德港上饶码头、上万高速公路建设。继续抓好320国道城区段改建、上广和上铅快速通道、德上高速怀玉山和大茅山连接线建设。开工建设上饶东、德兴两个500千伏变电站。完成农村公路硬化1500公里，新建、改造桥梁25座。大力推行政府与社会资本合作（PPP）等多种投融资模式，引导和撬动民间资本参与重大基础设施建设。

增强中心城区带动力。按照区域中心城市、高铁枢纽城市、优秀旅游城市和田园化城市的要求，尽快修编中心城市总体规划。拉开高铁新区、空港新区、西货站物流园区“三个新区”建设框架，重点建设高铁新区、西货站物流园区。改造凤凰大道绿化景观带，将上饶大道、广信大道、凤凰大道、五三大道打造成中心城区绿化景观轴。改造五三大道、中山路、信阳路店招门楣，打造商业精品街。加快新火车站综合交通枢纽、大坳引水城西支线工程（二期）、机场大道、稼轩大道、上饶大道北延伸、滨江大道西延伸等城市续建项目建设步伐，开工建设市城乡规划展示馆、市青少年综合实践基地及市体校、市中心医院及康复中心、市金融中心、城东路堤防洪工程、夜市夜宵一条街等城建项目。继续推进行政区划调整。加强城市管理，打造“精细优美”城市。深入开展全国文明城市、全国卫生城市、国家森林城市创建工作。

加快城镇群建设。编制“1+5”信江河谷城镇群、环鄱阳湖城镇群、德兴婺源生态城镇群等“三群”城镇化发展规划及下一层级的产业发展、旅游、生态环境保护及重大基础设施专项规划。强化城镇群动态管理，逐步实现重大项目“规划一张图、审批一支笔、管理一部法”。强化产城融合，推动符合条件的开发区向城市功能区转型。培育壮大一批商贸重镇、文化名镇、旅游强镇、特色小镇。

提升城乡信息化服务水平。深入推进信息惠民国家试点城市和“宽带中国”示范城市建设。继续加快4G网络建设，力争4G网络覆盖全市80%的行政村和主要交通干线。

（七）以培育特色产业集群为抓手，发展壮大县域经济，努力在打造县域经济升级版上取得新突破

加大对县域经济的扶持力度。全面落实“权力下放、政策松绑、服务到位”要求，全力支持县域经济发展。积极争取更多项目纳入鄱阳湖生态经济区、原中央苏区振兴发展规划和扶贫攻坚等国家扶持计划，积极争取省政府支持赣东北扩大开放合作加快发展的扶持政策，继续落实好市委、市政府全力支持广丰德兴跻身全国百强县（市）、全力支持婺源三清山创建全国一流景区、全力支持“1+5”信江河谷城镇群建设、全力支持鄱余万创建全国优质农产品种养加工基地建设的扶持政策。

加快县域经济发展升级。加强对县域产业的规划和指导，实施园区提升计划，按照“找准支柱、做强支点、夯实支撑、优化支持”的要求，加快培育形成特色产业集群。坚持以城镇化为载体，引导人口有序集聚和资源要素合理配置，促进城乡一体化发展。大力推进“三产”融合式发展。大力支持省直管县改革，赋予省直管县更多的发展自主权，增强发展活力。 （八）以改善民生为根本，全力抓好民生事业，努力在打造更高水平的“幸福上饶”上取得新突破

深入推进生态文明建设。全面落实省政府《关于建设生态文明先行示范区的实施意见》，力争在建设生态文明先行示范区进程中走在全省前列。加快编制生态环境保护和建设总体方案。继续抓好再生资源循环经济产业园建设。强力推进污染减排工作，继续抓好中心城区和县城污水管网建设，确保“十二五”规划中四项主要污染物减排任务如期完成、重点减排项目如期建成、城镇污水处理厂稳定运行。加快工业园区污水处理厂建设，力争2016年全市所有工业园区污水处理厂建成投运。启动上饶市垃圾焚烧发电项目前期工作。抓好废弃矿山生态修复和土壤重金属污染治理工程。推进资源节约集约利用。积极推进造林向营林转变，完成造林27万亩。

加快实施重大民生工程。落实更加积极的就业创业政策，确保新增城镇就业4.7万人，新增转移农村劳动力7.2万人。加大精准扶贫力度，力争全年脱贫8.2万人，完成扶贫移民搬迁6500人。提高企业退休人员基本养老金、城乡居民基础养老金、城乡低保、重点优抚对象等生活补助标准。做好机关事业单位养老保险制度改革工作。继续做好医疗保险大病关爱工作。落实和完善医疗保险付费总额控制，推动支付方式改革。开工建设各类保障房3.7万套。加强全市饮用水源保护，推进鄱阳湖水环境治理工程，建设农村饮水工程208处，解决76万农村人口饮水安全问题。加快推进市群艺馆、市博物馆、市老年福利中心等重大民生项目建设。

大力发展社会事业。完成1105所农村义务教育学校达标建设。大力发展现代职业教育。加快推进上饶幼儿师范高等专科学校筹建工作。支持上饶师范学院转型发展和婺源茶校筹建茶文化高专学校。推进文化精品创作，扶持赣剧、弋阳腔、婺源傩舞等传统文化发展。完成婺源县、德兴市基本公共文化服务标准化、均等化建设试点工作。完成直播卫星“户户通”工程，推动广播电视全覆盖。办好鄱阳湖中华龙舟赛、鄱阳湖国际自行车大赛、玉山首届中式台球世界锦标赛等赛事活动。稳步推进县级公立医院综合改革，巩固完善基本药物制度，新农合参合率稳定在98%以上，筹资水平提高到450元。严格食品药品监管，确保“舌尖上的安全”。加强关心下一代工作，引导青少年健康成长。积极应对人口老龄化，大力发展养老服务产业。稳定低生育水平，降低出生人口性别比，提高出生人口素质。做好双拥工作，争创新一轮“双拥模范城”。加强国防动员，做好优抚安置工作。切实做好防汛、防火、防震减灾工作，提高气象防灾减灾能力。发展社会慈善事业，营造良好公益环境。

加强社会治理体系建设。扎实推进法治上饶建设，组织引导基层群众依法参与社会治理。严格落实安全生产主体责任，全面做好安全生产、道路交通、消防等安全工作，坚决防止重特大安全事故发生。扎实推进平安上饶建设，加强辅警力量配备，加大打防管控力度，保持全市良好的治安秩序。推进依法治访，注重用法律手段解决涉法涉诉信访问题。强化应急管理体系建设，加快形成统一指挥、反应灵敏、功能齐全、协调有序、运转高效的应急管理机制。

各位代表：科学编制“十三五”规划，是今年一项十分重要的任务。我们将认真总结“十二五”规划执行情况，加强调研论证，突出重大项目，科学编制全市“十三五”发展规划，力求使规划与国家产业政策和投资重点相衔接，与全省区域发展战略相衔接，与全市发展定位相衔接，力争更多项目进入国家和省里的“十三五”规划盘子。

各位代表！

建设法治政府，是贯彻依法治国基本方略、推进法治上饶建设的重要举措。我们将紧扣保障社会公正、促进社会诚信、维护社会秩序三大重点任务，加快建设职能科学、权责法定、执法严明、公开公正、廉洁高效、守法诚信的法治政府。

坚持依法决策。在重大决策中，严格执行公众参与、专家论证、风险评估、合法性审查、集体讨论决策等法定程序，确保决策制度科学、程序正当、过程公开、责任明确。进一步建立健全听证制度、决策咨询制度、重大行政决策合法性审查制度、决策终身责任追究制度和责任倒查制度，用制度的严肃性体现决策的责任性，用程序的完整性保障决策的科学性。全力做好立法人才的引进和培养工作，确保在省人大批准我市享有立法权时能高效开展工作。

强化用权监督。主动接受人大的法律监督、工作监督和政协的民主监督，强化监察、审计等内部监督，让权力在阳光下运行。自觉接受舆论监督和社会监督，充分运用市长热线、公众微信等通讯平台，听取群众意见建议。对财政资金分配使用、国有资产监管、政府投资、政府采购、公共资源转让、公共工程建设等权力集中的部门和岗位实行分事行权、分岗设权、分级授权，定期轮岗，强化内部流程控制，防止权力滥用。

建设诚信政府。全面推进政务公开，坚持以公开为常态、不公开为例外原则，推进决策公开、执行公开、管理公开、服务公开、结果公开，增强政府公信力。将政务履约和守诺服务纳入政府绩效评价体系，推动各地、各部门逐步建立健全政务和行政承诺考核制度。深入开展社会主义核心价值观以及公务员诚信、守法和道德教育，完善公务员诚信约束和问责机制。

优化政务环境。重点打击破坏经济发展环境的人和事，铁心硬手解决“小鬼难缠”的问题。加大行政不作为、慢作为、乱作为整治力度，使各级干部适应在约束中工作、习惯在监督下干事。全面落实廉政建设责任制，深入落实中央“八项规定”，持之以恒纠正“四风”，严肃查处各类违纪违法案件，强化源头治腐，切实做到干部清正、政府清廉、政治清明。

各位代表！上饶正处在发展升级、小康提速、绿色崛起的关键时期，我们的任务艰巨而繁重，使命重大而光荣。让我们更加紧密地团结在以习近平同志为核心的党中央周围，在省委、省政府和市委的坚强领导下，进一步解放思想、坚定信心、开拓创新、真抓实干，为建设四省交界现代化区域中心城市和江西绿色崛起重要增长极而努力奋斗！
